# The isolation and molecular characterization of *Mycobacterium avium* subsp. *paratuberculosis* in Shandong province, China

**DOI:** 10.1186/s13099-016-0092-6

**Published:** 2016-03-22

**Authors:** Ruichao Yue, Chunfa Liu, Paul Barrow, Fei Liu, Yongyong Cui, Lifeng Yang, Deming Zhao, Xiangmei Zhou

**Affiliations:** State Key Laboratories for Agrobiotechnology, Key Laboratory of Animal Epidemiology and Zoonosis, Ministry of Agriculture, National Animal Transmissible Spongiform Encephalopathy Laboratory, College of Veterinary Medicine, China Agricultural University, Beijing, 100193 China; School of Veterinary Medicine and Science, University of Nottingham, Sutton Bonington Campus, Loughborough, Leicestershire LE12 5RD UK; Institute of Microbiology, Chinese Academy of Sciences, Beijing, 100101 China

**Keywords:** *Mycobacterium avium* subspecies *paratuberculosis*, Molecular characterization, Prevalence, China

## Abstract

**Background:**

*Mycobacterium avium* subspecies *paratuberculosis* (Map) causes Johne’s disease in domestic and wild ruminants. It has been a debate that whether Map can cause Crohn’s disease in human. To our knowledge there is no report about molecular characterization of Map in China, although several Map strains have been reported in other country. The objectives of this study was to know the recent prevalence of Johne’s disease in dairy farms in Shandong province, and have a better understanding of genotypic distribution of Map in China.

**Methods:**

Johne’s disease was detected from 1038 individuals in 19 dairy farms by ELISA. Map in fecal and milk specimens was identified by Ziehl-Neelsen staining and confirmed using PCR-REA. In addition, frozen sections of ileum and mesenteric lymph nodes from two Map shedding cows were performed to observe the histopathological changes. Next-generation sequencing technology was performed to get whole genome sequences.

**Result:**

A total of 121 (11.7 %) animals were positive for Map antibody from 1038 sera tested, and 11 (57.9 %) dairy herds were positive for Map antibody. Typically histopathologic changes were observed in mesenteric lymph nodes. We have successfully isolated two Map strains, which both were Map-C. The current genome-wide analysis showed that the genome size of our isolates are respectively 4,750,273 and 4,727,050 bp with a same G + C content of 69.3 %, and the numbers of single nucleotide polymorphisms (SNPs) against Map K-10 are respectively 292 and 296.

**Conclusion:**

Map is a prevalent pathogen among dairy cattle in China. This study successfully isolated two Map strains from one Chinese dairy herd with signs of diarrhoea, and identified that the two isolates were both Map-C. Furthermore, these isolates were most closely related to Map K-10.

## Background

Paratuberculosis, also known as Johne’s disease, caused by *Mycobacterium avium* subspecies *paratuberculosis* (Map), is an important and highly prevalent disease of domestic and wild ruminants manifest as a chronic granulomatous enteritis with decreased milk production and in more serious cases leading to progressive emaciation and death. The disease is transmitted via milk and colostrum to calves and by the fecal-oral route to animals of all ages. Intra-uterine transmission can also occur and Map can also be detected in the saliva of cows, indicating this as a potential further mode of transmission [1, 2]. The disease is endemic in many parts of the world with serious economic losses which in the USA, for example, are estimated at between $200 and $250 million annually [3].

Map belongs to the *M. avium* complex [4] and consists of three important types designated “Sheep-type” (type S) and “Cattle-type” (type C) which were originally isolated from sheep or cattle respectively [5] and “Bison-type” (B-type) [6] all of which can be differentiated by IS1311 restriction endonuclease genotyping. Despite designation of strains as B, S or C there is no host-species specificity [7] and strains isolated from sheep or cattle can be either C or S type [8]. Most of the ruminant population in India are infected by Map ‘B type’ strains [9, 10].

The importance of Map as a zoonotic pathogen has not yet been clarified [11, 12]. The clinical presentation and pathology of Crohn’s disease (CD) bears more than a superficial resemblance to paratuberculosis. Despite some studies indicating no absolute relationship between Map and CD [13, 14] a proportion of individuals with CD contain Map or Map DNA in their blood or infected intestinal tissue [15]. However, Map can be isolated from stool samples of individuals who are healthy or presenting with unrelated diseases [16]. This latter observation indicates the general susceptibility of humans to Map infection. Map can be readily isolated from milk [17], including pasteurized milk [18] and may also be isolated from the muscles of infected cattle, consumption of which, may be an additional potential route of transmission to humans [19].

Complete eradication of the disease from cattle, although desirable, is extremely difficult [20]. Vaccination can be effective to control clinical disease, reducing faecal shedding and increasing productivity but it does not completely eliminate infection [21, 22]. Various quarantine measures can be adopted at dairy farms for controlling this disease including the isolation of young calves from infected cattle. However, young stock may also excrete Map in their feces emphasizing current difficulties in disease control.

For molecular identification of Map by PCR a number of different targets have been used including f57 [23], HspX [24], genes 251 and 255 [25], ISMav2 [26], ISMpa1 [27] and ISMAP02 [28], some of which (f57, HspX and ISMAP02) are unique to Map but which are present in low (1–6) copy number with associated limited potential for differentiation [29]. For this reason IS900, with 14–18 copies is a preferable PCR target [30].

Additional PCR targets may be identified from Map genome sequences. Currently, the only fully annotated genome is that of Map strain K-10 [31] although additional draft sequences from strains ATCC 19698, Map s397 and Map S5 from different countries have been published on NCBI. Despite the likely high prevalence of paratuberculosis in China, no genome sequences are available for any Chinese strains. Although Map has been isolated from Chinese farms in the past [32], no information is available on the strain types and molecular characterisation present. The present study thus had the following objectives: (1) investigating the prevalence rate of Map infection in Chinese farms with clinical signs of diarrhoea, (2) typing of Map strains by PCR-restriction endonuclease analysis based on polymorphisms in IS1311 and, (3) to sequence the whole genome to contribute to a better understanding of the evolution of Map.

## Methods

### Sample collection

1038 individual bovine serum samples were collected between October 2014 and August 2015 from 19 different dairy farms located in nine distinct geographic regions of Shandong province. The herds were selected at random and varied between 300–800 animals. Individual cows showing diarrhoea were selected in addition to some animals showing no clinical signs. Approximately 10 % of the animals were selected all of them being adult cows over 24 months of age. Milk, faeces and tissues were sampled from the herd showing the highest frequency of positive sera. All the faecal samples were obtained via per-rectal drags, and were kept at 4 °C up to 48 h prior to processing for culture and DNA extraction. Milk samples were collected in 50-ml tubes after four initial strippings were discarded. Ileum and mesenteric lymph nodes were collected from two euthanized cows with positive antibody for Map. All protocols and procedures were performed according to the Chinese Regulations of Laboratory Animals—The Guidelines for the Care of Laboratory Animals (Ministry of Science and Technology of People’s Republic of China) and Laboratory Animal Requirements of Environment and Housing Facilities (GB 14925–2010, National Laboratory Animal Standardization Technical Committee). The license number associated with their research protocol was 20110611–01 and the animal study proposal was approved by The Laboratory Animal Ethical Committee of China Agricultural University.

### Detection of Map antibody

In order to identify the herd with highest frequency of positive sera the 1038 sera were tested for the presence of antibodies to Map using a commercial antibody test kit (IDEXX). Sera with S/P ratios ≤0.45 and ≥0.55 were considered negative and positive, respectively. Intermediate S/P values were considered “suspect” and regarded as negative for the data analysis.

### Detection of bacteria

10 mg feces without any treatment was taken to spread evenly on to the surface of a clear glass slide with 20 ml water drop. For milk samples, there was a little difference. Centrifugation was needed to get more bacterium. After centrifugation at a speed of 12000 rpm for 5 min, the bottom precipitate was collected as the samples for smear, and the same procedure was used as that of faecal samples. Tissues were used as frozen sections. Slides were stained using the Ziehl–Neelsen method and examined by light microscopy for the presence of acid-fast bacilli. Map presence was confirmed by PCR amplification of IS*900* as described previously [33].

### Sample preparation and culture

A modification of the double centrifugation methods was used to cultivate Map [34]. Briefly, two grams of faeces were added to 35 ml of sterile distilled water. Samples tubes were vortexed for 1 min and then allowed to stand undisturbed for 45 min. Five ml of the supernatant were added to 25 ml of 0.9 % hexadecylpyridinium chloride (HPC) and then vortexed for 2 min and allowed to stand undisturbed for 12 h at room temperature. Tubes were centrifuged at 1500*g* for 30 min, the supernatant was discarded and the pellet was resuspended in 1 ml of antibiotic mixture (50 µg/ml amphotericin B, and 100 µg/ml nalidixic acid, and 100 µg/ml vancomycin). This was mixed well and incubated for 12 h at 37 °C. Herrold’s Egg Yolk Agar with mycobactin J and ANV (BD, USA) were inoculated with 0.2 ml of the suspension and incubated at 37 °C for 6 weeks.

### Isolation and identification of Map and strain typing of isolates

Colonies that appeared within 1 week were discarded and slower-growing colonies with typical colonial morphology were checked for acid fast bacteria, and then detected by PCR based on IS900 as mentioned above. In order to generate pure and sufficient biomass, a single colony of each confirmed Map isolate was subcultured into 7H9 broth supplemented with OADC and Mycobactin J and incubated at 37 °C. The culture was reconfirmed as Map by PCR and the purity checked by light microscopy with Ziehl–Neelsen method staining. The strain type was identified by IS*1311* PCR and restriction endonuclease analysis (REA) using the primers and method described previously [33]. The identification as *M. avium* subsp. *paratuberculosis* was confirmed by PCR using IS*900* and REA using *Alw*I digestion. The IS*1311* PCR-REA was used to distinguish C strains from S strains using *Hinf*I and *Mse*I (details shown in Table 1). PCR primers are detailed in Table 2.

**Table 1:** Details of PCR-REA

PCR	Target	Forward primer	Reverse primer	Predicted product size (bp)	Enzyme	subspecies	Predicted band sizes (bp)
1	IS900	P90	P91	413	*Alw*I	Map	147,266
2	IS1311	M56	M119	608	*Hinf*I/*Mse*I	Map S	285,323
						Map C	67,218,285,323
						Map B	67,218,323
						*M. avium* subsp. *avium*	134,189,285

**Table 2:** Sequence of the primers

Primer	Sequence
P90	GAA GGG TGT TCG GGG CCG TCG CTT AGG
P91	GGC GTT GAG GTC GAT CGC CCA CGT GAC
M56	GCG TGA GGC TCT GTG GTG AA
M119	ATG ACG ACC GCT TGG GAG AC

### Next-generation whole genome sequencing and analysis

Massive parallel sequencing using the Solexa-technology (Illumina) was performed at the Chinese Academy of Sciences. Two isolates were sequenced using the Illumina HiSeq 2500 platform using one flow cell lane with 125-cycle paired end chemistry. Short reads were assembled using SOAPdenovo (http://www.soap.genomics.org.cn), a genome assembler developed specifically for next-generation short-read sequences [35]. The SOAP GapCloser was also used to close gaps. Reads were mapped by using BWA [36], and SNPs and indels were called and filtered by SAM tools [37]. A rooted tree was computed using MEGA. Phylogenetic tree was calculated using the maximum likelihood method by whole genome alignment and performing 1000 bootstraps replicates. Several references are selected for comparation, include type C, type B, and type S (Table 3).

**Table 3:** A list of accession numbers for organisms used in this paper

Organisms	Accession number	Type
Map K-10	NC_002944.2	C
Map ATCC 19698	NZ_AGAR00000000.1	C
Map s397	AFIF00000000	S
Map JQ5	NZ_AHAZ00000000.1	S
Map S5	NZ_ANPD01000178.1	B
Map 10-4404	NZ_AYNR00000000.1	B
Map 2015WD-1	LKUS00000000	C
Map 2015WD-2	LKUT00000000	C

### Statistical analysis

Statistical comparisons between milk detection and fecal detection were determined using the Chi square test with p < 0.05 denoting statistical significance. SPSS software (Chicago, IL, USA) was used.

## Results

### The survey of the sera

Screening of 1038 serum samples from 19 dairy farms by the *Mycobacterium paratuberculosis* antibody test kit detected 121 (11.7 %) animals positive for Map antibody, and 11 (57.9 %) dairy farms were positive for Map antibody. Of 11 dairy farms with signs of diarrhoea, 7 (63.6 %) were positive and of 8 dairy farms with no signs of diarrhea, 3 (37.5 %) were positive. The highest positive rate within these herds was 78.9 % and this herd was selected for isolation and identification of Map (Table 4).

**Table 4:** Results of herd analysis for Map antibody

Dairy farm	Total number	sampling number	The number of cow with diarrhoea	The number of positive	The number of negative	Positive rate (%)
1	435	40	0	3	37	7.50
2	560	50	1	0	50	0
3	480	50	3	7	43	14.0
4	584	60	0	0	60	0
5	300	30	0	0	30	0
6	650	65	3	0	65	0
7	628	60	0	5	55	8.33
8	400	40	0	0	40	0
9	550	55	5	14	41	25.5
10	740	70	4	0	70	0
11	800	80	6	0	80	0
12	360	36	4	12	24	33.3
13	720	70	0	0	70	0
14	450	45	6	17	28	37.8
15	620	62	2	8	54	12.9
16	780	78	1	8	70	10.3
17	800	80	3	30	50	37.5
18	300	19	8	15	4	78.9
19	480	48	0	2	46	4.17
Total number	10,637	1038	46	121	917	11.7

### The morphology of bacteria and pathological changes in the tissues

**Fig. 1:**
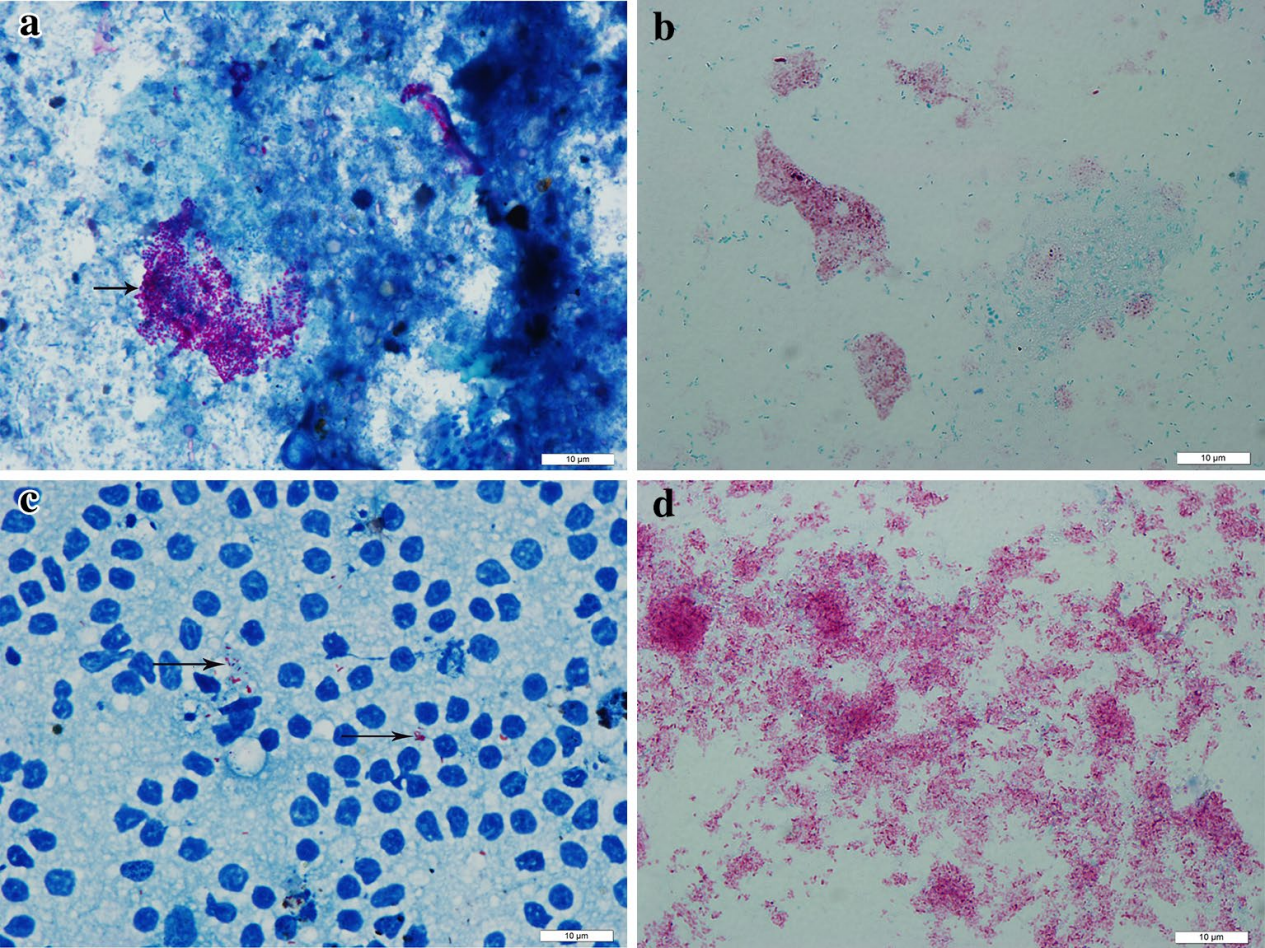
Ziehl–Neelsen staining for bacilli. **a** Faecal samples showing positive for Ziehl–Neelsen stain (*arrow*). **b** Milk samples showing positive for acid-fast staining (*arrows*). **c** Ziehl–Neelsen stained impression smears of the mesenteric lymph nodes showed large numbers of acid-fast bacilli (*arrows*). **d** Large numbers of clustered acid-fast bacilli were observed in the smears taken from a purified colony (*arrows*)

**Fig. 2:**
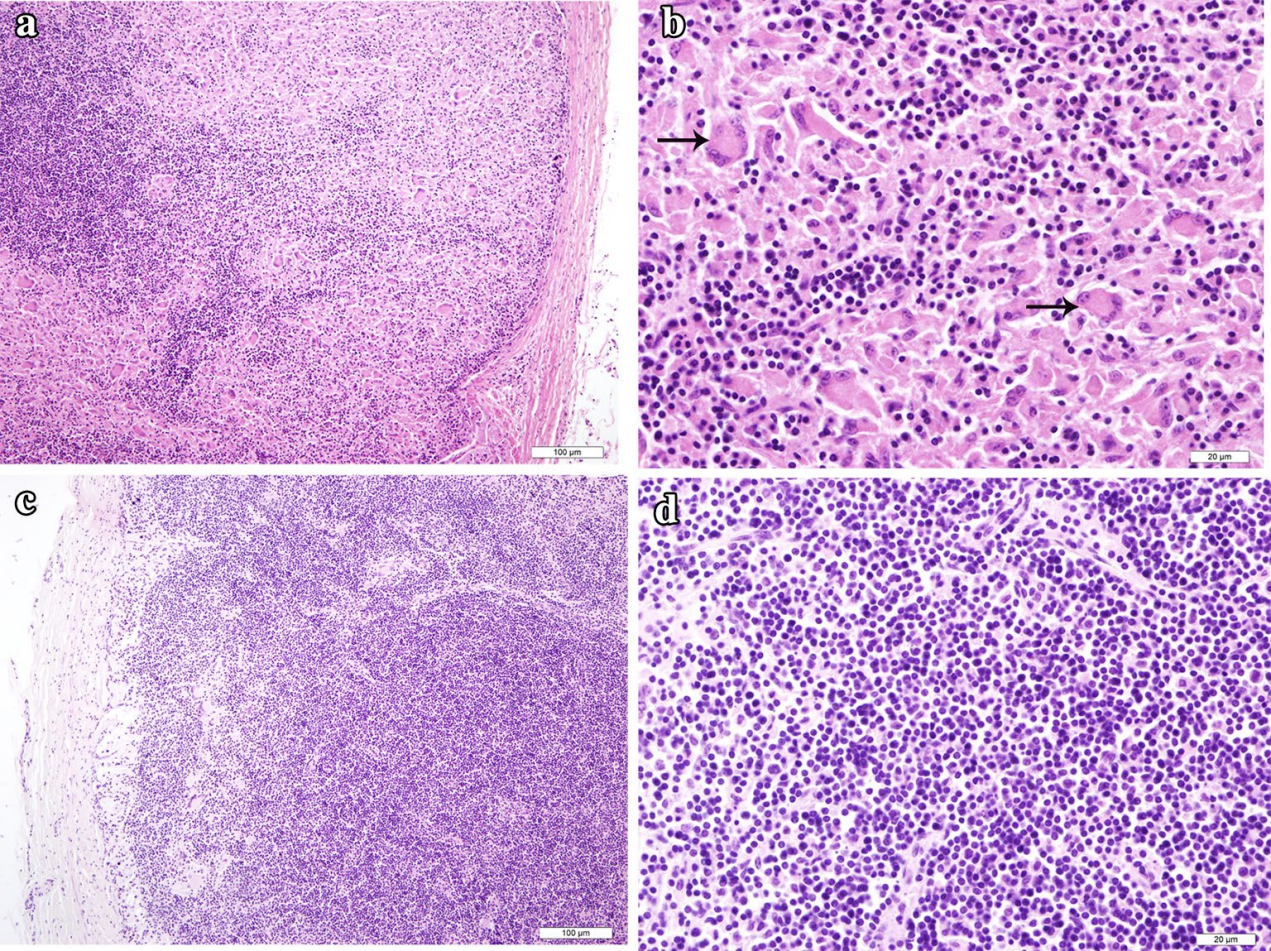
Histopathological observation of mesenteric lymph nodes. **a** A representative of lymph node thin section stained with H&E showing the decrease of original lymphocytes and the increase of reticular cells and epithelioid cells. **b** The amplification of *panel* **a**, showing the characteristic diffuse granulomas in which epithelioid cells, macrophages, multinucleated giant cells (*arrows*) and lymphocytes aggregated. **c** The normal histology structure of mesenteric lymph nodes. **d** The amplification of *panel* **d**

Nineteen faecal and milk samples were sampled for Ziehl–Neelsen staining to detect individual cattle positive for acid fast bacilli. Of these 13 (68.4 %), 6 (31.6 %) faecal samples and milk samples respectively were positive for acid-fast bacilli (Fig. 1a, b). We euthanized two cattle with diarrhoea, emaciation and reduced milk production. Postmortem examination showed gross lesions in the mesenteric lymph nodes showing 5–6 fold enlargement compared with normal mesenteric lymph nodes (date not shown). However, there were no obvious gross lesions in the gastro-intestinal tract. Histopathological examination of mesenteric lymph nodes showed the characteristic diffuse granulomas with epithelioid cells, multinucleated giant cells and macrophages (Fig. 2a, b) compared with normal mesenteric lymph nodes(Fig. 2c, d). However, no granulomas were observed in the ileum (date not shown). Ziehl–Neelsen stained impression smears of the mesenteric lymph nodes showed large numbers of acid-fast bacilli (Fig. 1c). Large numbers of clustered acid-fast bacilli were observed in the smears taken from a purified colony (Fig. 1d).

### The identification and strain typing of MAP

**Fig. 3:**
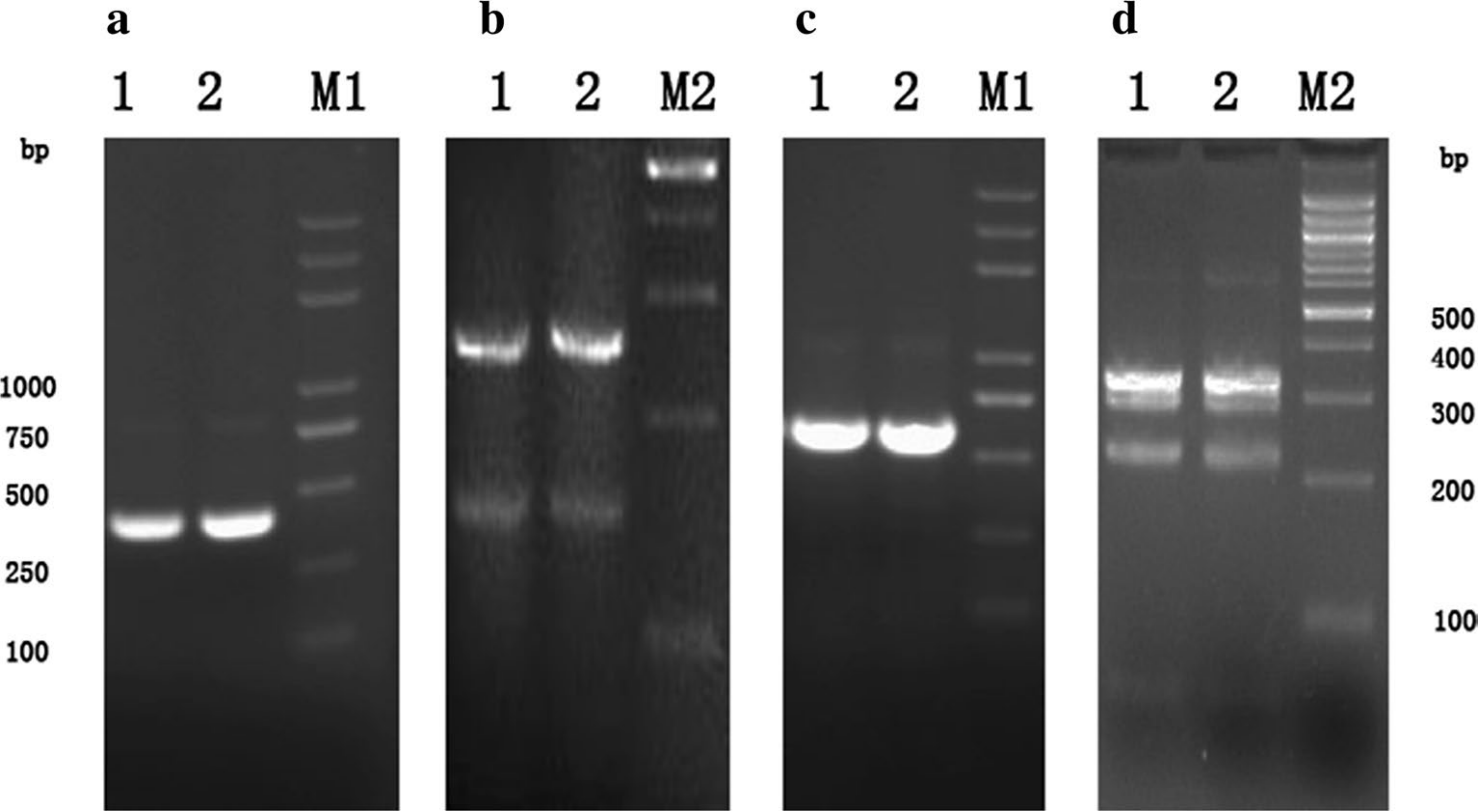
The results of identification and strain typing of MAP using PCR-REA. **a** PCR amplification of IS900 gene segment. *Lanes* *M1* DL2000 Plus Marker; *1* 2015WD-1; *2* 2015WD-2. **b** *Alw*I restriction endonuclease analysis of reaction IS900. *Lane* *M2*100 bp DNA ladder, *Lanes* *1*, *2* in panel **b**, **c**, **d** are all identical to those in *panel* **a**. **c** PCR amplification of IS1311 gene segment. *Lane M1* DL2000 Plus Marker. **d** *Hinf*I and *Mse*I restriction endonuclease analysis of reaction IS1311, *Lane M2* 100 bp DNA ladder

The PCR products are shown Fig. 3. Two isolates were both identified as positive using the IS*900* PCR. We obtained the target fragment, whose size was 413 bp (Fig. 3a) which was digested using restriction endonuclease *Alw*I which resulted in two bands, one at 147 bp, and other at 266 bp (Fig. 3b). For further identification of strain type, IS*1311* PCR was conducted and followed by digestion by two restriction endonucleases (*Hinf*I/*Mse*I). The results showed that both isolates were identified as positive with the target band of 608 bp (Fig. 3c). Restriction endonuclease analysis of the IS1311 PCR reaction products confirmed the presence of bands of 67, 218, 285 and 323 bp (Fig. 3d), which indicated that both isolates were type C.

### Whole genome sequencing and phylogenetic analysis

**Fig. 4:**
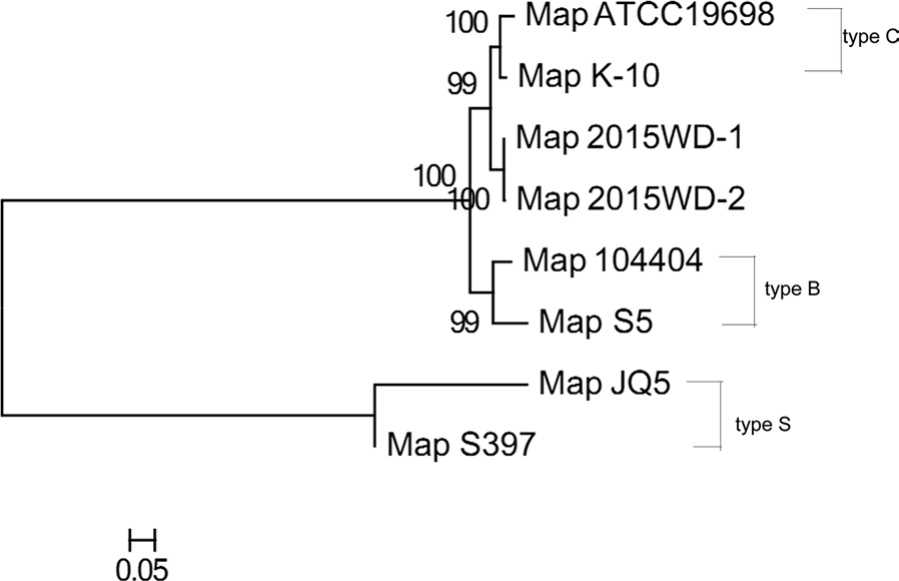
Phylogenetic relationships of Map strains based on whole genome sequences. The tree was constructed by the maximum-likelihood method. *Scale bar* indicates nucleotides substitutions per site

Although we grouped the two isolates, it was not enough to deeper understand the genomic differences between our isolates and reference strains. Here Next-generation sequencing technology (Illumina, HiSeq 2500) was used to sequence the whole genome of the two isolates from cattles in China. As a result, we successfully got two draft sequences named 2015WD-1 and 2015WD-2, the accession numbers are LKUS00000000 and LKUT00000000, respectively. The results showed that the genome size of 2015WD-1 and 2015WD-2 are respectively 475,0273 and 472,7050 bp with a same G + C content of 69.3 %. The numbers of single nucleotide polymorphisms (SNPs) in our Map isolates against Map K-10 are respectively 292 and 296. Also, 84 and 96 indels are found in our isolates against Map K-10. More information between our isolates and reference strains are presented in Table 5. In the phylogenetic tree (Fig. 4) based on the nucleotide sequences, we can see our two isolates are most closely relate to type C representative strains Map K-10 and Map ATCC19698, and farthest from type S representive strains Map JQ5 and Map s397.

**Table 5:** Summary of next generation sequencing results

Parameters	0908	1026
Genome size (bp)	4,750,273	4,727,050
% G + C	69.3 %	69.3 %
Sequencing depth (X)	210	150
Mean paried read distance	438	459
No. of indels against K-10	84	96
No. of SNPs againt K-10	292	296
No. of indels against s397	338	390
No. of SNPs againt s397	3681	3705
No. of indels against s5	622	847
No. of SNPs againt s5	1016	984
% Homology to K-10	99.89	99.86
% Homology to s397	99.75	99.76
% Homology to s5	99.87	99.85

## Discussion

The first study on paratuberculosis in China was recorded in Inner Mongolia in 1953, and then it was reported consistently in China [38]. In Shandong province, there has been one previous study of the prevalence of paratuberculosis. This indicated a prevalence of 29.34 % in 2011 and 14.93 % in 2012, detected by the interferon gamma release assay [39]. Detection by ELISA has indicated prevalences of between 2 and 4 % in Tibet, Shanghai and Guangxi provinces and highly variable values of 0–73.4 % in Inner Mongolia [40–42].

By contrast levels of infection in Europe and other western countries appear to be equally high with individual positive rates in cattle in estimated at 20 % and the herd-level prevalence at >50 % according to the summary of Nielsen and Toft [43]. Other studies showed seroprevalence rates of between 1.99 % in Asturias, Spain [44], 16.1 % in the east of Canada [45] and 29.8 % in Northern India [46]. In Egypt in 26 Holstein dairy herds in 2010 and 2013 herd and individual level prevalence were recorded as 65.4 % and 13.6 % respectively. [47]. No country claims that it is free on MAP.

Our studies showed a seroprevalence of 11.7 % in individual level in Shandong province of China, which is a little higher than most of provinces of China and a little lower than Europe, Indian and Canada. And the herd level prevalence was 57.9 % in 19 dairy herds we have investigated, indicated that Map has become a common pathogen in dairy farms in China. Among the 19 herds, there were more or less individuals behaved diarrhea, but the Map antibody positive rate were zero. Maybe these symptoms of diarrhea were caused by any other organisms than Map.

Two strains of Map were isolated from a farm showing heavy losses in their dairy cattle from diarrhoea resulting in weakness and reductions of milk production.

We successfully isolated two MAP from one serious loss of dairy farm in which many cattle behaved diarrhoea, weakness, reduction of milk production. Environmental bacteria such as streptococci were observed to be present in the samples (data not shown) but decontamination using an adaptation off two HPC methods ([34]; Report, 2011) was clearly successful.

Several methods were used to detect the pathogen; fast-acid staining showed that the isolation rate of milk (9/19) was less than in faeces (14/19), but the difference is not significant (p = 0.184). The histological picture and bacillary morphology were consistent with paratuberculosis.

IS1311 PCR-REA provides similar information as IS900 RFLP analysis but is more useful than RFLP analysis where DNA is degraded or present in low concentrations [48]. To further confirm identification we used the IS900 PCR-REA to confirm the identity of isolates as Map and IS1311 PCR-REA for strain typing use of which indicated that the isolates we obtained were Map and were C strains.

Most previous studies have indicated that C strains were the dominant strains in cattle in countries as different as Australia, New Zealand, South America, North America and Europe [5, 48–50]. The exception appears to be that B strains are the most prevalent (82 %) genotype of Map in all domestic ruminants in India with C strains present as a minority strain [51]. It was thus important to know which types existed in China. Although the present study involved two strains only these were both C strains, which is at least consistent with most other previous studies.

In terms of whole genome sequencing, our two isolates have less base pairs than Map K-10, but the same G + C content with Map K-10. Our two isolates have least number of SNPs (N = 292 for 2015WD-1 and 296 for 2015WD-2) and indels (N = 84 2015WD-1 and 96 for 2015WD-2) against Map K-10 when compared with Map s397 and Map s5, indicating that Chinese isolates are most likely originated from Map K-10 with few changes. From phylogenetic tree, we can also find that 2015WD-1and 2015WD-2 are most closely related to Map K-10, which further confirmed the identified strain type of our isolates.

Although there is no definite conclusion on the relationship between Map and CD, Map is detected sometimes in normal individuals, which less often than in Crohn’s patients [16]. The fact that Map can be found in normal individuals does not exclude it as the cause of the disease, because the normal individuals maybe subclinically infected. Perhaps CD is caused by complex factors. Map is one of them and other element such as environmental stress, immunosuppressive conditions and so on are involved.

## Conclusion

Map has become a common pathogen in dairy farms in China. Present study successfully isolated two Map strains from one Chinese dairy farm with signs of diarrhoea, and identified that the two isolates were both Map-C. Farther more, we found that our isolates were most closely relate to Map K-10.

## References

[1] Stevenson K (2015). Genetic diversity of *Mycobacterium avium* subspecies *paratuberculosis* and the influence of strain type on infection and pathogenesis: a review. Vet Res.

[2] Sorge US, Kurnick S, Sreevatsan S (2013). Detection of *Mycobacterium avium* subspecies *paratuberculosis* in the saliva of dairy cows: a pilot study. Vet Microbiol.

[3] Ott SL, Wells SJ, Wagner BA (1999). Herd-level economic losses associated with Johne’s disease on US dairy operations. Prev Vet Med.

[4] Thorel MF, Krichevsky M, Levy-Frebault VV (1990). Numerical taxonomy of mycobactin-dependent mycobacteria, emended description of *Mycobacterium avium*, and description of *Mycobacterium avium* subsp. avium subsp. nov., *Mycobacterium avium* subsp. *paratuberculosis* subsp. nov., and *Mycobacterium avium* subsp. silvaticum subsp. nov. Int J Syst Bacteriol.

[5] Collins DM, Gabric DM, de Lisle GW (1990). Identification of two groups of *Mycobacterium paratuberculosis* strains by restriction endonuclease analysis and DNA hybridization. J Clin Microbiol.

[6] Sohal JS, Singh SV, Singh PK, Singh AV (2010). On the evolution of ‘Indian Bison type’ strains of *Mycobacterium avium* subspecies *paratuberculosis*. Microbiol Res.

[7] Ronai Z, Csivincsik A, Gyuranecz M, Kreizinger Z, Dan A (2015). Molecular analysis and MIRU-VNTR typing of *Mycobacterium avium* subsp. *paratuberculosis* strains from various sources. J Appl Microbiol.

[8] Stevenson K, Hughes VM, de Juan L, Inglis NF, Wright F (2002). Molecular characterization of pigmented and nonpigmented isolates of *Mycobacterium avium* subsp. *paratuberculosis*. J Clin Microbiol.

[9] Sevilla I, Singh SV, Garrido JM, Aduriz G, Rodriguez S (2005). Molecular typing of *Mycobacterium avium* subspecies *paratuberculosis* strains from different hosts and regions. Rev Sci Tech.

[10] Yadav D, Singh SV, Singh AV, Sevilla I, Juste RA (2008). Pathogenic ‘Bison-type’ *Mycobacterium avium* subspecies *paratuberculosis* genotype characterized from riverine buffalo (Bubalus bubalis) in North India. Comp Immunol Microbiol Infect Dis.

[11] Waddell LA, Rajic A, Stark KD, Mc Es (2015). The zoonotic potential of *Mycobacterium avium* ssp. *paratuberculosis*: a systematic review and meta-analyses of the evidence. Epidemiol Infect.

[12] Rowbotham DS, Mapstone NP, Trejdosiewicz LK, Howdle PD, Quirke P (1995). *Mycobacterium paratuberculosis* DNA not detected in Crohn’s disease tissue by fluorescent polymerase chain reaction. Gut.

[13] Barrett JC, Hansoul S, Nicolae DL, Cho JH, Duerr RH (2008). Genome-wide association defines more than 30 distinct susceptibility loci for Crohn’s disease. Nat Genet.

[14] Over K, Crandall PG, O’Bryan CA, Ricke SC (2011). Current perspectives on *Mycobacterium avium* subsp. *paratuberculosis*, Johne’s disease, and Crohn’s disease: a review. Crit Rev Microbiol.

[15] Kirkwood CD, Wagner J, Boniface K, Vaughan J, Michalski WP (2009). *Mycobacterium avium* subspecies *paratuberculosis* in children with early-onset Crohn’s disease. Inflamm Bowel Dis.

[16] Tuci A, Tonon F, Castellani L, Sartini A, Roda G (2011). Fecal detection of *Mycobacterium avium* paratuberculosis using the IS900 DNA sequence in Crohn’s disease and ulcerative colitis patients and healthy subjects. Dig Dis Sci.

[17] Stabel JR, Bradner L, Robbe-Austerman S, Beitz DC (2014). Clinical disease and stage of lactation influence shedding of *Mycobacterium avium* subspecies *paratuberculosis* into milk and colostrum of naturally infected dairy cows. J Dairy Sci.

[18] Ellingson JL, Anderson JL, Koziczkowski JJ, Radcliff RP, Sloan SJ (2005). Detection of viable *Mycobacterium avium* subsp. *paratuberculosis* in retail pasteurized whole milk by two culture methods and PCR. J Food Prot.

[19] Alonso-Hearn M, Molina E, Geijo M, Vazquez P, Sevilla I (2009). Isolation of *Mycobacterium avium* subsp. *paratuberculosis* from muscle tissue of naturally infected cattle. Foodborne Pathog Dis.

[20] Wolf R, Orsel K, De Buck J, Barkema HW (2015). Calves shedding *Mycobacterium avium* subspecies *paratuberculosis* are common on infected dairy farms. Vet Res.

[21] Singh SV, Singh PK, Kumar N, Gupta S, Chaubey KK (2015). Evaluation of goat based ‘indigenous vaccine’ against bovine Johne’s disease in endemically infected native cattle herds. Indian J Exp Biol.

[22] Tewari D, Hovingh E, Linscott R, Martel E, Lawrence J (2014). *Mycobacterium avium* subsp. paratuberculosis antibody response, fecal shedding, and antibody cross-reactivity to Mycobacterium bovis in *M. avium* subsp. *paratuberculosis*-infected cattle herds vaccinated against Johne’s disease. Clin Vaccine Immunol.

[23] Vansnick E, De Rijk P, Vercammen F, Geysen D, Rigouts L (2004). Newly developed primers for the detection of *Mycobacterium avium* subspecies *paratuberculosis*. Vet Microbiol.

[24] Miller JM, Jenny AL, Ellingson JL (1999). Polymerase chain reaction identification of *Mycobacterium avium* in formalin-fixed, paraffin-embedded animal tissues. J Vet Diagn Invest.

[25] Bannantine JP, Baechler E, Zhang Q, Li L, Kapur V (2002). Genome scale comparison of *Mycobacterium avium* subsp. *paratuberculosis* with *Mycobacterium avium* subsp. avium reveals potential diagnostic sequences. J Clin Microbiol.

[26] Shin SJ, Chang YF, Huang C, Zhu J, Huang L (2004). Development of a polymerase chain reaction test to confirm *Mycobacterium avium* subsp. *paratuberculosis* in culture. J Vet Diagn Invest.

[27] Olsen I, Johansen TB, Billman-Jacobe H, Nilsen SF, Djonne B (2004). A novel IS element, ISMpa1, in *Mycobacterium avium* subsp. *paratuberculosis*. Vet Microbiol.

[28] Stabel JR, Bannantine JP (2005). Development of a nested PCR method targeting a unique multicopy element, ISMap02, for detection of *Mycobacterium avium* subsp. *paratuberculosis* in fecal samples. J Clin Microbiol.

[29] Castellanos E, de Juan L, Dominguez L, Aranaz A (2012). Progress in molecular typing of *Mycobacterium avium* subspecies *paratuberculosis*. Res Vet Sci.

[30] Bull TJ, Hermon-Taylor J, Pavlik I, El-Zaatari F, Tizard M (2000). Characterization of IS900 loci in *Mycobacterium avium* subsp. *paratuberculosis* and development of multiplex PCR typing. Microbiology.

[31] Li L, Bannantine JP, Zhang Q, Amonsin A, May BJ (2005). The complete genome sequence of *Mycobacterium avium* subspecies *paratuberculosis*. Proc Natl Acad Sci USA.

[32] Zhang J. Detecting pathogeny of bovine paratuberculosis [master degree]: Yunnan Agricultural University. 2008.

[33] Marsh I, Whittington R, Cousins D (1999). PCR-restriction endonuclease analysis for identification and strain typing of *Mycobacterium avium* subsp. *paratuberculosis* and *Mycobacterium avium* subsp. avium based on polymorphisms in IS1311. Mol Cell Probes.

[34] Douarre PE, Cashman W, Buckley J, Coffey A, O’Mahony JM. Isolation and detection of *Mycobacterium avium* subsp. *paratuberculosis* (MAP) from cattle in Ireland using both traditional culture and molecular based methods. Gut Pathog. 2010;2:11. 10.1186/1757-4749-2-11 PMC2954866 20875096

[35] Luo R, Liu B, Xie Y, Li Z, Huang W, et al. SOAPdenovo2: an empirically improved memory-efficient short-read de novo assembler. Gigascience.1:18. 10.1186/2047-217X-1-18 PMC3626529 23587118

[36] Li H, Durbin R (2009). Fast and accurate short read alignment with Burrows–Wheeler transform. Bioinformatics.

[37] Li H, Handsaker B, Wysoker A, Fennell T, Ruan J (2009). The sequence alignment/Map format and SAMtools. Bioinformatics.

[38] Cui Z. Paratuberculosis. Diseases of dairy cattle. 1 ed. China Agriculture Press. 2007. p. 220–3.

[39] Meng Han LZ, Wang Jilong, Ding Jiabo (2014). Epidemiological investigation of large-scale dairy farm on tuberculosis and paratuberculosis in Shandong province. Chin Agric Sci Bullet.

[40] Xiaoqiang Wang ZH, Liu Xinyu, Jiang Wenteng (2015). Seroprevalence of paratuberculosis in Yaks in some regions of Tibet, China. China Dairy Cow.

[41] Hui Zhai GF, Xiao gu, Dongrao Li. Serological investigation and prevention measures of cow paratuberculosis. China Livest Poult Ind. 2015:101–2.

[42] Bao Tian YB, Jiang Yonglu, Sun Qingyu (2014). Serological survey of paratuberculosis in cattle of some dairy farms in inner Mongolia. Anim Husb Feed Sci.

[43] Nielsen SS, Toft N (2009). A review of prevalences of paratuberculosis in farmed animals in Europe. Prev Vet Med.

[44] Gasteiner J, Wenzl H, Fuchs K, Jark U, Baumgartner W (1999). Serological cross-sectional study of paratuberculosis in cattle in Austria. Zentralbl Veterinarmed B.

[45] McKenna SL, Keefe GP, Barkema HW, McClure J, Vanleeuwen JA (2004). Cow-level prevalence of paratuberculosis in culled dairy cows in Atlantic Canada and Maine. J Dairy Sci.

[46] Singh SV, Singh AV, Singh R, Sharma S, Shukla N (2008). Sero-prevalence of bovine Johne’s disease in buffaloes and cattle population of North India using indigenous ELISA kit based on native *Mycobacterium avium* subspecies *paratuberculosis* ‘Bison type’ genotype of goat origin. Comp Immunol Microbiol Infect Dis.

[47] Amin AS, Hsu CY, Darwish SF, Ghosh P, AbdEl-Fatah EM (2015). Ecology and genomic features of infection with *Mycobacterium avium* subspecies *paratuberculosis* in Egypt. Microbiology.

[48] Whittington RJ, Hope AF, Marshall DJ, Taragel CA, Marsh I (2000). Molecular epidemiology of *Mycobacterium avium* subsp. *paratuberculosis*: IS900 restriction fragment length polymorphism and IS1311 polymorphism analyses of isolates from animals and a human in Australia. J Clin Microbiol.

[49] Whipple D, Kapke P, Vary C (1990). Identification of restriction fragment length polymorphisms in DNA from *Mycobacterium paratuberculosis*. J Clin Microbiol.

[50] Cousins DV, Williams SN, Hope A, Eamens GJ (2000). DNA fingerprinting of Australian isolates of *Mycobacterium avium* subsp *paratuberculosis* using IS900 RFLP. Aust Vet J.

[51] Kaur P, Filia G, Singh SV, Patil PK, Ravi Kumar GV (2011). Molecular epidemiology of *Mycobacterium avium* subspecies *paratuberculosis*: IS900 PCR identification and IS1311 polymorphism analysis from ruminants in the Punjab region of India. Comp Immunol Microbiol Infect Dis.

